# Rare Germline Pathogenic Variants Identified by Multigene Panel Testing and the Risk of Aggressive Prostate Cancer

**DOI:** 10.3390/cancers13071495

**Published:** 2021-03-24

**Authors:** Tú Nguyen-Dumont, James G. Dowty, Robert J. MacInnis, Jason A. Steen, Moeen Riaz, Pierre-Antoine Dugué, Anne-Laure Renault, Fleur Hammet, Maryam Mahmoodi, Derrick Theys, Helen Tsimiklis, Gianluca Severi, Damien Bolton, Paul Lacaze, Robert Sebra, Eric Schadt, John McNeil, Graham G. Giles, Roger L. Milne, Melissa C. Southey

**Affiliations:** 1 Precision Medicine, School of Clinical Sciences at Monash Health, Monash University, Melbourne, VIC 3168, Australia; tu.nguyen-dumont@monash.edu (T.N.-D.); Jason.Steen@monash.edu (J.A.S.); pierre-antoine.dugue@monash.edu (P.-A.D.); Anne-Laure.Renault@monash.edu (A.-L.R.); Fleur.Hammet@monash.edu (F.H.); Maryam.Mahmoodi@monash.edu (M.M.); Derrick.Theys@monash.edu (D.T.); Helen.Tsimiklis@monash.edu (H.T.); Graham.Giles@cancervic.org.au (G.G.G.); Roger.Milne@cancervic.org.au (R.L.M.); 2 Department of Clinical Pathology, Melbourne Medical School, The University of Melbourne, Melbourne, VIC 3010, Australia; 3 Centre for Epidemiology and Biostatistics, Melbourne School of Population and Global Health, The University of Melbourne, Melbourne, VIC 3010, Australia; jdowty@unimelb.edu.au (J.G.D.); Robert.MacInnis@cancervic.org.au (R.J.M.); 4 Cancer Epidemiology Division, Cancer Council Victoria, Melbourne, VIC 3004, Australia; 5 Department of Epidemiology and Preventive Medicine, School of Public Health and Preventive Medicine, Monash University, Melbourne, VIC 3004, Australia; Moeen.Riaz@monash.edu (M.R.); Paul.Lacaze@monash.edu (P.L.); John.McNeil@monash.edu (J.M.); 6 CESP Inserm U1018, Faculté de Médecine—Univ. Paris-Sud, Faculté de Médecine—UVSQ, Université Paris-Saclay, 94805 Villejuif, France; gianluca.severi@inserm.fr; 7 Gustave Roussy, 94805 Villejuif, France; 8 Department of Surgery, Austin Health, The University of Melbourne, Heidelberg, VIC 3084, Australia; damien.bolton@unimelb.edu.au; 9 Department of Genetics and Genomic Sciences, Icahn School of Medicine at Mount Sinai, New York, NY 10029, USA; robert.sebra@mssm.edu (R.S.); eric.schadt@mssm.edu (E.S.)

**Keywords:** aggressive prostate cancer, gene panel testing, predisposition, genetic risk factors

## Abstract

**Simple Summary:**

Identifying which men at the time of prostate cancer diagnosis have, or will progress to, an aggressive fatal disease will allow clinicians to assist men in making better informed treatment decisions. This will not only be important for those men whose disease is likely to remain indolent and who are currently undergoing unnecessary treatment procedures, but also for those who may need to be targeted with immediate and potentially life-saving therapy. Our case-control study confirms that men who carry *BRCA1*, *BRCA2* and *ATM* germline pathogenic variants are at increased risk of aggressive disease and provides risk estimates that will be used by clinicians to improve counselling.

**Abstract:**

While gene panel sequencing is becoming widely used for cancer risk prediction, its clinical utility with respect to predicting aggressive prostate cancer (PrCa) is limited by our current understanding of the genetic risk factors associated with predisposition to this potentially lethal disease phenotype. This study included 837 men diagnosed with aggressive PrCa and 7261 controls (unaffected men and men who did not meet criteria for aggressive PrCa). Rare germline pathogenic variants (including likely pathogenic variants) were identified by targeted sequencing of 26 known or putative cancer predisposition genes. We found that 85 (10%) men with aggressive PrCa and 265 (4%) controls carried a pathogenic variant (*p* < 0.0001). Aggressive PrCa odds ratios (ORs) were estimated using unconditional logistic regression. Increased risk of aggressive PrCa (OR (95% confidence interval)) was identified for pathogenic variants in *BRCA2* (5.8 (2.7–12.4)), *BRCA1* (5.5 (1.8–16.6)), and *ATM* (3.8 (1.6–9.1)). Our study provides further evidence that rare germline pathogenic variants in these genes are associated with increased risk of this aggressive, clinically relevant subset of PrCa. These rare genetic variants could be incorporated into risk prediction models to improve their precision to identify men at highest risk of aggressive prostate cancer and be used to identify men with newly diagnosed prostate cancer who require urgent treatment.

## 1. Introduction

While age, family history, and ethnicity are well-established risk factors for prostate cancer, genetic factors also play an important role and are estimated to account for 57% of its heritability [1]. Genome-wide association studies have thus far identified 269 common variants accounting for about 34% of the familial relative risk [2]. Linkage studies have identified a missense substitution in *HOXB13* associated with increased risk of early-onset prostate cancer [3,4]. Rare variants in several genes that were initially implicated in risk for breast or ovarian cancer predisposition (e.g., *BRCA1* [5], *BRCA2* [6,7], *CHEK2* [8], *BRIP1* [5], and *ATM* [9]), as well as the mismatch repair (MMR) genes [10], have also been reported to increase the risk of prostate cancer. Candidate gene approaches, including those using whole-exome sequencing, have contributed to our understanding of genetic risk factors for prostate cancer. For instance, Schaid et al. performed whole-exome sequencing on highly selected prostate cancer cases (*n* = 491) and controls (*n* = 429) followed by targeted sequencing of candidate susceptibility genes in an independent dataset comprising 2917 cases and 1899 controls. Eleven genes previously associated with increased risk of prostate cancer (including *ATM*, *BRCA2*, and *HOXB13*) were identified along with ten new candidate genes [11].

The prevalence of germline pathogenic variants in men diagnosed with prostate cancer has been predominantly investigated for DNA repair genes and reported to range between 7.5% and 19% [12,13,14,15,16]. However, these findings have limited clinical utility if they cannot distinguish men whose prostate cancer is likely to remain indolent from those who may need to be targeted with immediate and potentially life-saving therapy. A growing number of studies have therefore used a case-case design to address this issue and aimed to identify germline variants that can distinguish those prostate cancers that will develop into aggressive, clinically relevant disease.

Pritchard et al. reported that germline pathogenic variants in DNA-repair genes were more frequent in men with metastatic prostate cancer than in men with localized disease (82/692, 11.8% and 23/499, 4.6%, respectively; *p* < 0.001), with the highest number of pathogenic variants being identified in *BRCA2* (5.3%), *ATM* (1.6%), and *CHEK2* (1.9%) in men with metastatic disease [13]. Mijuskovic et al. applied the BROCA panel to screen 139 metastatic cases of prostate cancer and 141 indolent cases. They reported a higher frequency of germline protein-truncating variants in men with metastatic disease (*n* = 17/139, 12.2%) compared with men with indolent disease (*n* = 4/141, 2.8%) (*p* = 0.004). The genes containing the highest number of protein-truncating variants in men with metastatic prostate cancer were *BRCA2*, *ATM*, and *NBN* [14].

We recently reported a case-case study of prostate cancer in which we compared the prevalence of germline pathogenic variants in 787 men with aggressive disease and 769 with non-aggressive disease [17]. We found that the proportion of men with aggressive prostate cancer who carried a *BRCA2* pathogenic variant exceeded that observed in men with non-aggressive prostate cancer (18/787 carriers, 2.3% and 4/769 carriers, 0.5%, respectively; *p* = 0.004). We observed a higher proportion of men with aggressive prostate cancer carrying pathogenic variants in *ATM* than that in men with non-aggressive prostate cancer (14/787 carriers, 0.02% and 5/769 carriers, 0.01%, respectively), although the difference did not reach statistical significance (*p* = 0.06) [17]. In another case-case study, Darst et al. assessed 2770 men with aggressive and 2775 men with non-aggressive prostate cancer cases from 12 international studies and found that risk for aggressive prostate cancer was associated with rare pathogenic variants in *BRCA2* (odds ratio (OR), 95% confidence interval: OR = 3.19, 1.94–5.25, *p* < 0.001), *PALB2* (OR = 6.31, 1.83–21.68, *p* < 0.001), and *ATM* (OR = 1.88, 1.10–3.22, *p* = 0.02) [18].

The possible association between *CHEK2* germline pathogenic variants and risk of prostate cancer still requires clarification due to the few and conflicting reports to date. Our case-case study identified 10 (1.3%) men with *CHEK2* pathogenic variants and aggressive disease and 5 (0.7%) with the same variants but non-aggressive disease (*p* = 0.30) [18]. Wu et al. reported a higher proportion of *CHEK2* c.1100delC carriers in men with lethal prostate cancer (1.28%) compared with those with low-risk disease (0.16%) [19], but Leongamornlert et al. observed that only “non-1100delC” protein-truncating variants contributed to the aggressive form of the disease [12].

Many commercial laboratories offer clinical genetic testing for hereditary cancer syndromes using panels that range from small cancer syndrome-specific gene panels, based on guidelines, to comprehensive, pan-cancer panels. Some clinical laboratories, such as Ambry Genetics, Invitae, and GeneDx, offer prostate cancer-specific panels that include the following genes: *ATM*, *BRCA1*, *BRCA2*, *CHEK2*, *HOXB13*, *MLH1*, *MSH2*, *MSH6*, *NBN*, *PALB2*, *PMS2*, and *TP53.* Evidence is emerging that genetic information can guide treatment modalities, hence the need to better understand genetic risk factors associated with the risk of aggressive prostate cancer.

In this study, we performed targeted sequencing of 26 genes commonly included on panel tests for cancer predisposition. We defined aggressive prostate cancer as fatal prostate cancer or prostate cancer that met criteria described by Hurwitz et al. [20], i.e., diagnosis of prostate cancer of category T4, N1, or M1 or a Gleason score of 8 or greater. Controls in our study were men who had not been diagnosed with aggressive prostate cancer, i.e., men unaffected with prostate cancer and men with prostate cancer that was not aggressive by the above definition (effectively treating aggressive and non-aggressive prostate cancer as separate diseases).

## 2. Results

### 2.1. Prevalence of Germline Pathogenic Variants in Men with Aggressive Prostate Cancer

There were a total of 89 germline pathogenic variants in 85/837 (10%) men with aggressive prostate cancer (individual variant details are provided in Supplementary Table S1).

Of these 89 pathogenic variants, 28 were nonsense, 31 frameshits, 4 inframe deletions, 8 splicing, and 18 missense variants. Pathogenic variants were identified in *BRCA2* (number of carriers; prevalence: 20; 2.4%), *ATM* (16; 1.8%), *HOXB13* (12; 1.4%), and *CHEK2* (9, 1.1%). There were five carriers (0.6%) of germline pathogenic variants in each of *BRCA1*, *FANCM*, and *RNASEL*. There were four or less carriers of a pathogenic variant in *PALB2*, *MSH6*, *RECQL*, *PMS2*, *BARD1*, *BRIP1*, *MSH2*, *NBN*, and *RAD51D*. All the pathogenic *MUTYH* variants were monoallelic. No pathogenic variants were identified in *CDH1*, *MLH1*, *MRE11A*, *NF1*, *PTEN*, *RAD50*, *RAD51C*, *TP53*, and *STK11*.

Four men were found to carry more than one germline pathogenic variant. One carried two pathogenic *BRCA2* variants known to be in cis and was previously identified by us [17]. One carrier of *BRCA2* c.6486_6489del; p.Lys2162Asnfs*5 was found to carry *RECQL* c.1859C > G; p.Ser620*. One carrier of *CHEK2* c.1100delC; p.Thr367Metfs*15 also carried *FANCM* c.5101C > T; p.Gln1701*. One carrier of *ATM* c.709dup; p.Thr237Asnfs*17 also carried the nonsense variant *HOXB1*3 c.327C > G; p.Tyr109* (also reported in [17]).

### 2.2. Associations between Germline Pathogenic Variants and Aggressive Prostate Cancer

**Figure 1:**
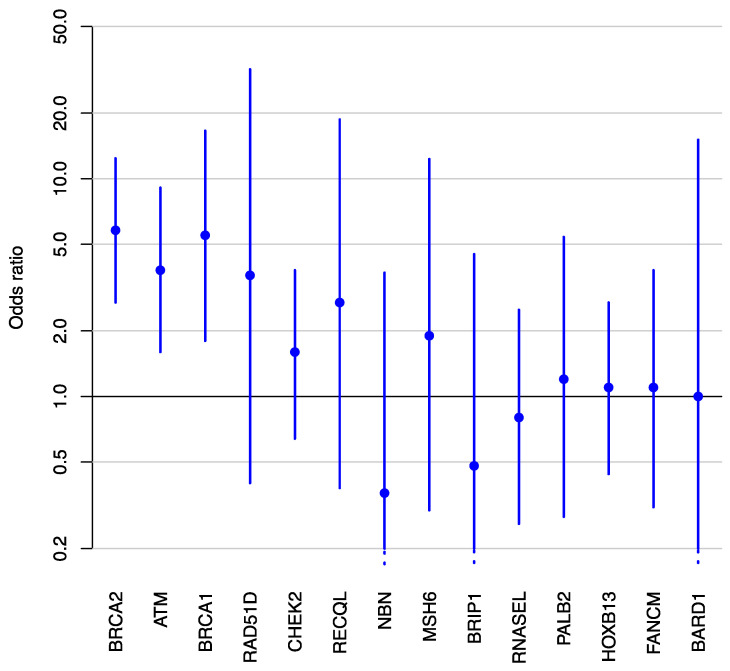
Adjusted odds ratios (large dots) and corresponding 95% confidence intervals (vertical lines) for the association between aggressive prostate cancer and germline pathogenic variants in various genes, sorted by *p*-value.

Gene-panel testing of the controls (i.e., men who did not meet criteria for aggressive prostate cancer) identified 273 germline pathogenic variants in 265/7261 (4%) men (Supplementary Table S2), which represents a lower prevalence of pathogenic variants than in aggressive prostate cancer cases (*p* < 0.0001). After excluding a small number of men with pathogenic variants in more than one gene, a total of 833 cases and 7255 controls were eligible for a case-control analysis. Table 1 presents the clinical characteristics of the men diagnosed with aggressive prostate cancer (cases) included in the statistical analysis. The estimated age-adjusted ORs are presented in Table 2 and Figure 1. We found evidence of an association between aggressive prostate cancer and three genes, with estimated ORs of 5.8 (95% confidence interval (CI): 2.7–12.4) for *BRCA2*, 5.5 (1.8–16.6) for *BRCA1*, and 3.8 (1.6–9.1) for *ATM*. The same three genes were associated with aggressiveness of prostate cancer in a case-case analysis (Supplementary Table S3) but *BRCA1* and *ATM* were not in an analysis where men with non-aggressive prostate cancer were excluded (Supplementary Table S4). Sensitivity analyses showed that the main results were almost unchanged by the inclusion of the small number of men with pathogenic variants in multiple genes (Supplementary Table S5).

## 3. Discussion

Although the recognition of an important subset of prostate cancer that is aggressive and clinically relevant is not new, there has not been, until very recently, a common evidence-based definition of aggressive prostate cancer. The literature, including reports from epidemiological studies, have used various combinations of clinical parameters, making it difficult to compare and combine studies. In this study, we used the definition recently published by Hurwitz et al., and the hard end point of prostate cancer death to facilitate further elucidation of prostate cancer etiology, including its genetic risk factors, and advance the prevention strategies specifically targeting aggressive prostate cancer [20].

We observed that 10% (85/837) of men with aggressive prostate cancer carried a germline pathogenic variant in a gene commonly included on panel tests for cancer predispostion, more than the 4% (265/7261) of men without aggressive prostate cancer (*p* < 0.0001). Consistent with the literature, we found that pathogenic variants in *BRCA2* and *BRCA1* are associated with increased risk of aggressive disease with ORs of 5.8 (2.7–12.4) and 5.5 (1.8–16.6), respectively. The identification of men carrying pathogenic variants in these genes has therapeutic relevance. For instance, PARP inhibitors have been shown to induce substantial responses in patients with metastatic prostate cancer expressing homologous recombination DNA-repair defects [21]. These tumors also appear to be responsive to platinum-based chemotherapy [22], consistent with what has been shown for breast and ovarian cancer diagnosed in women who carry a germline pathogenic variant in *BRCA1* and *BRCA2*. The identification of a germline pathogenic variant also provides information that is highly relevant to relatives, both men and women, as cascade testing can inform risk management strategies for family members.

By combining data from 13 research studies, representing 5560 men with prostate cancer and 3353 unaffected controls, the PRACTICAL consortium conducted the largest gene sequencing study of *ATM* and estimated an OR for overall prostate cancer risk of 4.4 (2.0–9.5) for pathogenic *ATM* variant carriers [9]. The PRACTICAL study included 1313 men with prostate cancer whose sequencing data are also included in the present report. Although their definition of “aggressiveness” was similar, the PRACTICAL report also defined sub-groups of aggressiveness: “non-aggressive” (stage T1–T2 and Gleason score 6 disease and, if deceased, death was not due to prostate cancer) and “intermediate aggressive” (those who did not fulfill the criteria for aggressive or non-aggressive disease). When comparing men with aggressive prostate cancer and unaffected controls and men with aggressive and non-aggressive disease, PRACTICAL estimated an OR of 5.4 (2.4–12.5, *p* < 0.001) and 1.6 (0.9–3.0, *p* = 0.135), respectively. In our study, the OR associated with risk of aggressive prostate cancer was 3.8 (1.6–9.1) when using men who did not have prostate cancer or did not meet our criteria for aggressive prostate cancer as controls.

One limitation of our study was the fact that the cases and controls were not age-matched. All ORs were adjusted for age, minimizing the impact of this age difference, but we cannot rule out residual confounding or other subtle biases due to this age difference. Another limitation was that our treatment of missing data in the definition of aggressive prostate cancer was conservative, and could cause a small downward bias in our OR estimates. In addition, our approach to targeted sequencing was limited to the coding regions and proximal intron-exon junctions of the genes included on the panel and could only detect single nucleotide variants and small insertions and deletions. Additionally, our analyses focused on germline variants identified as pathogenic in ClinVar. Our findings may thus underestimate the true prevalence of pathogenic missense variants, especially in genes that are less extensively studied than *BRCA1* and *BRCA2*, for which a database of reclassified variants has been established [23].

The strengths of our study include the use of a control dataset recruited from the same country as the cases, for which individual- and variant-level information was available. This is in contrast to other studies including Pritchard et al., who compared the pathogenic variant carrier prevalence of their studies with the ExAC public database. From a bioinformatics processing point of view, although the ideal case-control study design would involve generating sequencing data from the same sequencing platform and applying a single common bioinformatics pipeline [24,25], this is in reality very difficult to achieve, especially in studies requiring large sample sizes to have sufficient power to detect any effect. In our study, although different sequencing platforms were used to generate the raw sequencing data, we reduced potential artefactual variant calls by utilizing the processing pipeline that was the most appropriate for the sequencing technology used to produce the raw sequencing data for the case and the control subjects, then harmonizing the variant calls by (i) restricting calls to regions that are equally able to be called across the three targeted regions and (ii) applying the same filtering and annotation pipelines.

Despite a growing recognition of the role of rare missense variants in cancer predisposition, especially in breast cancer and for genes such as *CHEK2* [26] and *ATM* [27], missense variants individually are currently most commonly classified clinically as variants of uncertain significance. Functional assays have the potential to contribute to the evidence required for interpreting this category of variants, as recently demonstrated with *PALB2* [28,29,30,31]. The emerging functional evidence is being incorporated into international efforts aimed at defining the magnitude of cancer risk associated with specific missense variants in cancer predisposition genes and by ClinGen expert panels to support clinical classification of missense variants.

## 4. Materials and Methods

### 4.1. Study Subjects

Participants to this study came from (i) the Melbourne Collaborative Cohort Study (MCCS), (ii) the Aggressive Prostate Cancer (APC) study, (iii) the Risk Factors for Prostate Cancer Study (RFPCS), (iv) the Early-Onset Prostate Cancer Family Study (EOPCFS), and (v) the ASPirin in Reducing Events in the Elderly (ASPREE) study. The MCCS, APC, RFPCS, and EOPCFS are Australian research studies of prostate cancer that have been described previously [32,33,34]. The ASPREE study is a randomized, placebo-controlled trial for daily low-dose aspirin. Participants were Australians aged 70 years or older who had no previous diagnosis or current symptoms of atherothrombotic cardiovascular disease, physical disability, or dementia, and no current diagnosis of life-threatening cancer at enrolment. Study design [35], recruitment [36], baseline characteristics [37], and outcomes [38] have been previously described. Our statistical analysis only used ASPREE data that were collected at baseline.

Cases were men diagnosed with aggressive prostate cancer, which was defined to be fatal prostate cancer or prostate cancer that met the criteria described by Hurwitz et al. [20]: cancers that are clinical or pathologic category T4, N1, or M1 or Gleason score greater than or equal to 8. In this definition, missing criteria were assumed to be equal to the lower risk category, e.g., a missing Gleason score was taken to be 7 or lower. A total of 837 aggressive prostate cancer cases were identified from the MCCS, APC, RFPCS, and EOPCFS.

Controls were men who had not been diagnosed with aggressive prostate cancer, i.e., men unaffected by prostate cancer or whose prostate cancer did not meet the above criteria. There were 1238 men identified from MCCS, APC, RFPCS, and EOPCFS who did not have aggressive prostate cancer. Pathology data and precise ages at diagnosis were unavailable for ASPREE participants at baseline, so we excluded ASPREE men with a personal history of aggressive prostate cancer (defined, for the purpose of this exclusion criterion, to be men who died from prostate cancer or had metastatic prostate cancer), leaving a total of 6023 ASPREE controls.

### 4.2. Gene-Panel Testing

We analyzed rare genetic variants identified in the germline DNA of 837 cases (diagnosed with aggressive prostate cancer) and 7261 controls (not diagnosed with aggressive prostate cancer). Our analysis was restricted to the coding region and proximal intron-exon junctions of 26 genes (*ATM*: NM_000051, *BARD1*: NM_000465.2, *BRCA1*: NM_007294.3, *BRCA2*: NM_000059.3, *BRIP1*: NM_032043.2, *CDH1*: NM_004360.3, *CHEK2*: NM_007194.3, *FANCM*: NM_020937.2, *HOXB13:* NM_006361.5, *MLH1*: NM_000249.3, *MRE11A*: NM_005591.3, *MSH2*: NM_000251.2, *MSH6*: NM_000179.2, *MUTYH*: NM_001128425.1, *NBN*: NM_002485.4, *NF1*: NM_000267.3, *PALB2*: NM_024675.3, PMS2: NM_000535.5, *PTEN*: NM_000314.4, *RAD50*: NM_005732.3, *RAD51C*: NM_058216.2, *RAD51D*: NM_002878.3, *RNASEL*: NM_021133.3, *RECQL*: NM_002907.3, *STK11*: NM_000455.4, *TP53*: NM_000546.5).

Gene-panel testing had been previously performed for the ASPREE participants [39]. The ASPREE subjects were sequenced using an AmpliSeq panel on the Ion Torrent S5TM XL (Thermo Fisher Scientific, Waltham, MA, USA) and aligned sequencing files (BAMs) were provided for variant calling in this study. Of the 2075 participants from the MCCS, APC, RFPCFS, and EOPCFS, 1553 had been previously tested and reported in a case-case study of prostate cancer aggressiveness and a case-control study focused on *ATM* [9,18]. Additional gene-panel testing was performed for an additional 522 participants, using methods described previously [18]. Briefly, the germline DNA of these participants was sequenced in-house using a Hi-Plex panel on the NextSeq550 (Illumina, San Diego, CA, USA) [40].

### 4.3. Variant Calling and Filtering

Reads were mapped to the reference genome (GRch37). Variant calling was performed using VarDict 1.7 [41] and restricted to the overlap of the regions targeted by the two panels. For ASPREE controls sequenced on the Ion Torrent platform, variant calling had also been performed using the Torrent Variant Calling Suite v1.5 as previously described [39] and the intersection with the variant calls from VarDict was used in downstream analyses. Subsequent genetic analyses were restricted to variants with a minimum read depth 50× and variant allele frequency of 0.2. Additionally, for the ASPREE samples, we determined a conservative but high-confidence call set by filtering out variants present in more than 0.05% of all ASPREE participants (*n* = 65) and variants that had passed our quality filters described above in less than 95% of the calls at a given genomic location.

Variant annotation was performed using VarSeq VSClinical v2.2 (Golden Helix Inc., Bozeman, MT, USA) and included ClinVar annotations from November 2020. This study focused on rare, predicted, protein-truncating variants (PTVs) and pathogenic variants (including likely pathogenic variants). Rare variants were defined as those identified in ExAC v.0.3 with a minor allele frequency ≤0.01 in the non-cancer, non-Finnish European population (NFE non-TCGA). Genetic variants were considered pathogenic if they were annotated as “Pathogenic” or “Likely Pathogenic” in ClinVar. Mono-allelic pathogenic *MUTYH* variant carriers are reported in Supplementary Table S1 but were not included in our analysis. Predicted PTVs that were classified as “Conflicting” in ClinVar with annotations tending towards pathogenicity (e.g., *CHEK2*:c.1100delC) were included in this analysis. Also included were PTVs that were absent (unreported) from ClinVar, except if they were located in the last coding exon.

### 4.4. Statistical Analyses

For each of the genes considered, germline pathogenic variants were combined and an overall OR for their association with aggressive prostate cancer was estimated using unconditional multivariate logistic regression. These analyses were all adjusted for age, the only known prostate cancer risk factor that was available in all our datasets, where the age used was at baseline for ASPREE men and at prostate cancer diagnosis for men in the other studies. Men with prostate cancer that was not aggressive by the above definition were treated as controls for the main analyses since excluding men with non-aggressive disease would mean we could only make inferences about the population of men without non-aggressive prostate cancer.

Excluded from all analyses were women, and male participants in ASPREE with a personal history of aggressive prostate cancer (see Section 4.1) or with no genetic data. A small number of men with germline pathogenic variants in more than one gene were excluded from the main analyses. The effect on our results of this and other analytical choices was investigated with sensitivity analyses. Wald confidence intervals were calculated for each OR, and the likelihood ratio test was used to generate *p*-values for comparing nested models. All *p*-values were two-sided and a *p*-value threshold of 0.05 was used to define statistical significance. Fisher’s exact test was used to test for an overall difference in the prevalence of germline pathogenic variants in men with and without aggressive prostate cancer. All analyses were performed using R version 3.6.1 [42].

## 5. Conclusions

The cancer risks associated with many of the genes included in prostate cancer susceptibility gene panels are currently not well characterized. Further studies are required to generate the evidence base required for the clinical translation of gene-panel testing. Our study applied a new recommended definition of aggressive prostate cancer and provides further evidence that rare germline pathogenic variants in *ATM*, *BRCA1*, and *BRCA2* are associated with increased risk of this aggressive, clinically relevant subset of prostate cancer. These rare genetic variants could be incorporated into risk prediction models to improve their precision in identifying men at the highest risk of aggressive prostate cancer and identifying which men, at the time of diagnosis, require urgent treatment, while sparing patients at low risk from the morbidity associated with unnecessary treatment.

## Figures and Tables

**Table 1:** Characteristics of the men diagnosed with aggressive prostate cancer who were included in this study.

Variables and Values	Aggressive PrCa Cases, Number (%)
**Study**	non-missing = 833
ASPREE ^a^	0 (0%)
APC ^a^	322 (39%)
EOPCFS ^a^	185 (22%)
MCCS ^a^	140 (17%)
RFPCS ^a^	186 (22%)
**Age at diagnosis in years**	non-missing = 833
<60	258 (31%)
60–64	147 (18%)
65–69	262 (31%)
≥70	166 (20%)
**Gleason score**	non-missing = 628
2	1 (0%)
3	0 (0%)
4	5 (1%)
5	16 (3%)
6	41 (7%)
7	105 (17%)
8	189 (30%)
9	251 (40%)
10	20 (3%)
**Died from prostate cancer**	non-missing = 799
No	324 (41%)
Yes	475 (59%)

**^a^** ASPREE, ASPirin in Reducing Events in the Elderly study; APC, Aggressive Prostate Cancer study; EOPCFS, Early-Onset Prostate Cancer Family Study; MCCS, the Melbourne Collaborative Cohort Study; RFPCFS, Risk Factors for Prostate Cancer Study.

**Table 2:** Odds ratios (OR) and 95% confidence intervals (95%CI) for germline pathogenic ^a^ variants identified by panel testing of 26 genes in 833 men with aggressive prostate cancer (cases) and in 7255 men without aggressive prostate cancer (controls).

Gene	Cases (*n* = 833)		Controls ^b^ (*n* = 7255)		Adjusted OR (95% CI)	*p*-Value
	Number of Carriers	%	Number of Carriers	%		
*ATM*	14	1.7%	25	0.3%	3.8 (1.6–9.1)	0.0021
*BARD1*	1	0.1%	8	0.1%	1.0 (0.07–15.1)	0.97
*BRCA1*	5	0.6%	12	0.2%	5.5 (1.8–16.6)	0.0023
*BRCA2*	19	2.3%	24	0.3%	5.8 (2.7–12.4)	<0.0001
*BRIP1*	1	0.1%	14	0.2%	0.48 (0.05–4.5)	0.53
*CDH1*	0	0%	0	0%	-	-
*CHEK2*	8	1%	41	0.6%	1.6 (0.64–3.8)	0.32
*FANCM*	4	0.5%	23	0.3%	1.1 (0.31–3.8)	0.89
*HOXB13*	11	1.3%	18	0.2%	1.1 (0.44–2.7)	0.84
*MLH1*	0	0%	1	0%	-	-
*MRE11A*	0	0%	6	0.1%	-	-
*MSH2*	1	0.1%	0	0%	-	-
*MSH6*	3	0.4%	6	0.1%	1.9 (0.3–12.3)	0.49
*MUTYH*	0	0%	0	0%	-	-
*NBN*	1	0.1%	10	0.1%	0.36 (0.03–3.7)	0.39
*NF1*	0	0%	2	0%	-	-
*PALB2*	4	0.5%	13	0.2%	1.2 (0.28–5.4)	0.79
*PMS2*	2	0.2%	0	0%	-	-
*PTEN*	0	0%	0	0%	-	-
*RAD50*	0	0%	14	0.2%	-	-
*RAD51C*	0	0%	6	0.1%	-	-
*RAD51D*	1	0.1%	6	0.1%	3.6 (0.4–31.8)	0.25
*RECQL*	2	0.2%	5	0.1%	2.7 (0.38–18.7)	0.33
*RNASEL*	5	0.6%	23	0.3%	0.8 (0.26–2.5)	0.7
*STK11*	0	0%	0	0%	-	-
*TP53*	0	0%	2	0%	-	-
Total	82	9.8%	259	3.6%		

^a^ Pathogenic (including likely pathogenic) as defined by ClinVar and protein-truncating variants that are absent from ClinVar (accessed November 2020). Excludes protein-truncating variants located in the last coding exon and mono-allelic *MUTYH* pathogenic variants. For *PMS2*, panel design avoided regions of homology with the pseudo-gene *PMS2CL* (as described previously [17]). ^b^ Men without aggressive prostate cancer.

## Data Availability

All germline pathogenic variants identified are reported in Supplementary Tables S1 and S2. Sequencing data can be provided upon request to the corresponding author.

## Supplementary Materials

The following are available online at https://www.mdpi.com/2072-6694/13/7/1495/s1, Table S1: Germline pathogenic variants identified by gene panel testing in men with aggressive prostate cancer; Table S2: Germline pathogenic variants identified by gene panel testing in men without aggressive prostate cancer; Table S3: A case-only analysis where the outcome is aggressive prostate cancer versus all other prostate cancer; Table S4: A sensitivity analysis where men with non-aggressive prostate cancer were excluded rather than treated as controls; Table S5: A sensitivity analysis where a small number of men with germline pathogenic variants in multiple genes were included.
